# Network Pharmacology and Experimental Validation Reveal Ganodermanontriol Modulates Pneumonia via TNF/NF‐κB/MAPKs Signaling Pathway

**DOI:** 10.1002/fsn3.70123

**Published:** 2025-03-25

**Authors:** Shizhan Deng, Dequan Zhong, Yonggan Dong, Yanan Qian, Biao Wang, Mengxue Hu, Meng Liu, Kemeng Tan, Chaojie Zhang, Heng Tang

**Affiliations:** ^1^ Wanbei Coal Electric Group General Hospital Suzhou Anhui China; ^2^ Department of Orthopedics and Traumatology, Orthopedic Trauma Faculty Henan University of Chinese Medicine Zhengzhou Henan China; ^3^ Wanbei Coal Electric Group General Hospital Affiliated to Bengbu Medical University Suzhou Anhui China

**Keywords:** *e*xperimental validation, *Ganoderma lucidum* (Leyss.Ex Fr.) karst, Ganodermanontriol, network pharmacology, pneumonia

## Abstract

*Ganoderma lucidum* (Leyss. ex Fr.) Karst, commonly known as Lingzhi, has long been employed in traditional Chinese medicine for its medicinal properties, particularly in alleviating respiratory issues like cough and asthma. Recognized both as a therapeutic agent and an edible supplement, Lingzhi is celebrated for its health‐promoting benefits. Despite its widespread use, the effectiveness of *G. lucidum* in treating pneumonia has not been extensively studied, highlighting the need for further research. This research aimed to evaluate the potential of *G. lucidum* in pneumonia treatment and to uncover the mechanisms behind its effects, specifically examining how its active constituents influence inflammatory pathways. The study utilized approaches such as network pharmacology, bioinformatics, molecular docking, and in vivo experiments. High‐performance liquid chromatography (HPLC) and liquid chromatography–mass spectrometry (LC–MS) analyses revealed eight triterpenoids in *G. lucidum* , with ganodermanontriol being the most prominent. Molecular docking studies anticipated the interactions between these compounds and target proteins, while in vivo experiments on pneumonia‐induced rat models assessed the efficacy of ganodermanontriol. Additionally, HPLC and LC–MS confirmed the presence of eight triterpenoids in the ethanol extract of *G. lucidum* , predominantly ganodermanontriol. Network pharmacology and molecular docking identified key genes—including TNF, EGFR, ESR1, HIF1A, HSP90AA1, and SRC—that played significant roles in the regulation of inflammatory pathways. In vivo results demonstrated that ganodermanontriol treatment mitigated lung tissue damage in rats with experimentally induced pneumonia by reducing the release of inflammatory mediators. Further mechanistic studies showed that ganodermanontriol downregulated TNF‐α and inhibited the NF‐κB/MAPKs signaling pathways. These findings suggested that ganodermanontriol holds promising potential as an anti‐inflammatory agent for pneumonia by targeting the TNF/NF‐κB/MAPKs signaling pathway, offering a novel therapeutic approach.

## Graphical abstract

Ganodermanontriol, the primary triterpenoid identified in Ganoderma lucidum, was evaluated for its efficacy against pneumonia through network pharmacology, molecular docking, and in vivo rat models. The compound significantly reduced lung tissue damage and inflammatory mediators by targeting the TNF/NF‐κB/MAPKs signaling pathways. These results indicate that ganodermanontriol holds promise as an effective anti‐inflammatory agent for pneumonia treatment.

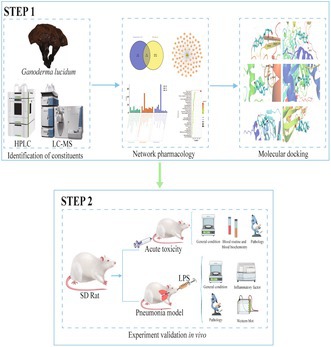

**Abbreviations**
ARDS: Acute respiratory distress syndrome
BP: biological process
CC: cellular component
COX‐2: Cyclooxygenase‐2
DAD: Diode‐array detector
DXMS: Dexamethasone‐treated group
GO: gene ontology
HE: Hematoxylin and eosin
HPLC: high‐performance liquid chromatography
IL‐1β: interleukin‐1 beta
IL‐6: interleukin‐6
iNOS: inducible nitric oxide synthase
LC–MS: liquid chromatography–mass spectrometry
MAPKs: mitogen‐activated protein kinases
MCC: maximal clique centrality
MF: molecular function
NF‐κB: nuclear factor kappa B
PPI: protein–protein interaction
TIC: total ion chromatograms
TNF: tumor necrosis factor (TNF)

## 1 Introduction

Pneumonia remains a widespread global health issue, ranking among the leading causes of morbidity and mortality worldwide (Lim 2022). It is defined by the inflammation of the lung parenchyma resulting from various causes, including bacterial, viral, and fungal infections, and involves intricate pathophysiological mechanisms (Ruan et al. 2023). The disease process results in damage to alveolar epithelial and pulmonary capillary endothelial cells, which increase capillary permeability and lead to pulmonary edema (Zhou et al. 2022). This cellular damage promotes excessive cytokine production, recruitment of leukocytes, and a substantial infiltration of inflammatory cells into the lung tissues (Sandrock and Norris 2015; Hu et al. 2022).

The inflammatory response in pneumonia is closely associated with the activation of several signaling pathways, particularly the tumor necrosis factor (TNF), nuclear factor kappa B (NF‐κB), and mitogen‐activated protein kinases (MAPKs) pathways (Lodi et al. 2025). Activation of these pathways triggered the transcription of pro‐inflammatory cytokines and chemokines, sustaining the inflammatory cascade and contributing to lung tissue damage (Li et al. 2024b). Current treatment approaches primarily rely on antimicrobial agents to eliminate the causative pathogens; however, they frequently do not address the underlying inflammatory processes (Jiang et al. 2024). Natural compounds have garnered attention for their potential therapeutic benefits due to their bioactive properties and lower rates of adverse effects (Naseem et al. 2025). *Ganoderma lucidum*, widely known as Lingzhi or Reishi, is a celebrated medicinal mushroom used in traditional Chinese medicine for over two millennia (Wang et al. 2020). Featured in the classic Chinese pharmacopeia Ben Cao Gang Mu, Lingzhi is valued for its therapeutic effects, including invigorating Qi (vital energy), soothing mental agitation, alleviating cough, and extending healthy lifespan (Liu et al. 2023). Contemporary scientific studies have demonstrated that *G. lucidum* is effective and safe in treating pulmonary diseases such as bronchial asthma and chronic bronchitis, highlighting its significant clinical application potential (Ming‐Chun et al. 2012; Rowaiye et al. 2023; Zhang et al. 2022). As both a medicinal and edible fungus, Lingzhi was widely used for its broad health benefits, particularly in China (Tang et al. 2017). Furthermore, the rise of antibiotic‐resistant strains emphasizes the urgent need for alternative therapies that can modulate the host's immune response and reduce inflammation (Bing et al. 2022).

*G. lucidum* contains bioactive components such as triterpenoids, polysaccharides, sesquiterpenoids, steroids, and alkaloids, with triterpenoids and polysaccharides (Umar et al. 2024a, 2024b; Li et al. 2018). Its anti‐inflammatory effects are primarily due to these triterpenoids and polysaccharides (Thuy et al. 2023; Łysakowska et al. 2023). Extensive research has shown that triterpenoids extracted from *G. lucidum* possess significant anti‐inflammatory properties through various complex mechanisms (Lin et al. 2016; Zeng et al. 2020). For example, methyl lucidenate L strongly inhibits LPS‐induced inflammation in macrophages by decreasing the expression of iNOS, COX‐2, and NF‐κB, and by blocking the phosphorylation of IκBα and IKKβ (Lou et al. 2024). Ganoderic acid A has been shown to regulate neuroinflammation in multiple sclerosis by increasing IL‐4 and BDNF expression, underscoring its potential as a treatment for neuroimmune disorders (Jia et al. 2021). Additionally, ganoderic acid D significantly lowers pro‐inflammatory cytokines such as TNF‐α, IFN‐γ, and IL‐17A in samples from Crohn's disease patients through the NF‐κB pathway, indicating its promise for managing inflammatory bowel diseases (Yuan et al. 2022). Ganodermanontriol is an important triterpenoid compound derived from *G. lucidum* , a well‐known medicinal mushroom (Peng et al. 2024) This compound has shown significant potential in various therapeutic areas, including anti‐tumor, neuroprotective, immune‐regulatory, anti‐lipid metabolism disorder, and liver protection applications, making it a promising candidate for multiple health‐related treatments (Huang et al. 2025). Formulations containing Lingzhi, like the coughing and gasping capsule, have shown remarkable effectiveness in treating respiratory disorders, achieving an overall effectiveness rate of 87.5% in chronic bronchitis patients without any reported toxic side effects (Liu et al. 2023). These results further endorse the potential of *G. lucidum* as a therapeutic agent for chronic respiratory illnesses (Kumar et al. 2024).

Nevertheless, the specific active substances and the underlying molecular mechanisms by which *G. lucidum* combats pneumonia remain unclear. Network pharmacology is an interdisciplinary approach that integrates systems biology, bioinformatics, and pharmacology to study the complex interactions between bioactive compounds, therapeutic targets, and disease pathways. Unlike traditional reductionist methods, it emphasizes a holistic view of drug actions by mapping multi‐component, multi‐target, and multi‐pathway relationships (Li et al. 2024a). It is particularly valuable for studying herbal medicines, such as *G. lucidum* , which contain diverse bioactive constituents with potentially interconnected roles (Wang, Wang, Hu et al. 2024). Network pharmacology bridges computational predictions (e.g., target screening, pathway enrichment) with experimental validation, enabling systematic exploration of how compounds modulate disease‐related pathways (Zhao et al. 2024). In this study, we employed HPLC and LC–MS to analyze the chemical composition of the ethanol extract of *G. lucidum* . Subsequently, the ganodermanontriol with the highest concentration was chosen for network pharmacology analysis, followed by experimental validation to examine the effects of *G. lucidum* on pneumonia. Our objective was to clarify how ganodermanontriol influences the TNF/NF‐κB/MAPKs signaling pathways to reduce pulmonary inflammation. Gaining an understanding of these mechanisms may offer valuable insights for developing ganodermanontriol as a novel therapeutic agent for pneumonia and potentially preventing its progression to acute respiratory distress syndrome.

## 2 Materials and Methods

### 2.1 Chemicals and Reagents

Dexamethasone and Lipopolysaccharide (LPS) were sourced from Sigma‐Aldrich (St. Louis, USA). Enzyme‐linked immunosorbent assay (ELISA) kits for measuring TNF‐α, IL‐6, and IL‐10 levels were acquired from Wuhan ColorfulGene Biological Technology (Wuhan, China). Rabbit primary antibodies targeting p‐p38, Erk1/2, p‐Erk1/2, JNK1/2/3, p‐JNK1/2/3, and GAPDH, with catalog numbers ab5831, ab138492, ab280888, ab40855, ab267373, and ab68153 respectively, were obtained from Abcam (Cambridge, UK). Additionally, rabbit primary antibodies for TNF‐R, p‐NF‐κB, NF‐κB, and IκBα, identified by catalog numbers #3736, #3033, #8242, and #9242, were procured from Cell Signaling Technology (Boston, USA). Reference standards, including Ganoderiol D, Ganoderiol J, Ganoderiol A, Ganoderic acid DM, Ganodermatriol, Ganoderiol F, Ganoderiol B, and Ganodermic acid S, were purchased from Chengdu Mansite Company.

### 2.2 Preparation of Ethanol Extract of *G. lucidum*

*G. lucidum* was acquired from the Bozhou Chinese herbal medicine market in July 2023, and a voucher specimen (V20230701) has been archived at the Wanbei Coal Electric Group General Hospital. The extraction procedure for *G. lucidum* was adapted from a previously published study (Wei‐Ye et al. 2024). In summary, the dried powder of *G. lucidum* was soaked in 95% ethanol (v/v) at a ratio of 1:15 (w/v) for 24 h. This mixture then underwent three cycles of reflux extraction at 100°C. The obtained liquid was subsequently filtered, concentrated under reduced pressure, and then lyophilized. The final product was the ethanol extract of *G. lucidum* (GLA).

### 2.3 UHPLC–MS Analysis of GLA Component

Sample preparation for phytochemical component analysis was conducted following the protocols detailed in prior studies (Wang, Wang, Hu et al. 2024, Wang, Wang, Luo 2024). For the qualitative assessment of GLTs, the total triterpenoid content was quantified using methodologies described in earlier research. High‐performance liquid chromatography (HPLC) of GLA was carried out with a diode‐array detector (DAD) and C18 spherical columns (5 μm, 4.6 × 250 mm, Agilent, Santa Clara, CA). Specific conditions for the liquid chromatography process are provided in the (Table S1). For the quantitative evaluation, GLTs concentrations were determined using LC–MS/MS. The parameters for both liquid chromatography and mass spectrometry were aligned with those used in previous investigations, and the reference standards were derived from our earlier work (Want et al. 2013; Yong 2013; Zelena et al. 2009). Standard curves and high‐resolution mass spectra for the four triterpenes are illustrated in Figures S2 and S3.

### 2.4 Network Pharmacology Analysis

#### 2.4.1 Prediction of Target Components of Ganodermatriol

The structural details of ganodermatriol, including its SMILES notation and structured data files, were obtained from the PubChem database (https://pubchem.ncbi.nlm.nih.gov/). These structural data were then used to identify potential targets using the Swiss Target Prediction tool (http://www.swisstargetprediction.ch/). Subsequently, the predicted targets were confirmed through the UniProt database (https://www.uniprot.org), leading to the establishment of the final list of drug targets.

#### 2.4.2 Pneumonia Target Acquisition

Relevant targets for treating pneumonia were obtained from the GeneCards and OMIM databases by searching the term “pneumonia.” These pneumonia‐associated targets were subsequently compared with the predicted targets of ganodermatriol using Venny 2.1.0 (https://bioinfogp.cnb.csic.es) to identify overlapping targets (Amberger et al. 2015; Safran et al. 2010; Shang et al. 2023).

#### 2.4.3 PPI Network Construction

The common targets of ganodermatriol associated with pneumonia were uploaded to the STRING database (Von Mering et al. 2003), utilizing a high confidence threshold of 0.700 for the analysis. The resulting TSV file was subsequently downloaded and imported into Cytoscape software version 3.9.1 to visualize the protein–protein interaction (PPI) network (Otasek et al. 2019). Centrality metrics of the network nodes were evaluated using the CytoNCA plugin available in Cytoscape's APPs section, facilitating the identification of key core targets (Tang et al. 2015).

#### 2.4.4 GO And KEGG Analysis

For Gene Ontology (GO) and Kyoto Encyclopedia of Genes and Genomes (KEGG) pathway analyses, the pneumonia‐associated targets of ganodermatriol were submitted to the Metascape database for enrichment analysis (Zhou et al. 2019). The GO enrichment encompassed biological processes (BP), cellular components (CC), and molecular functions (MF) to predict the functional distribution of the molecular targets. Additionally, KEGG pathway enrichment was conducted to identify pertinent signaling pathways linked to these targets. The top 10 results from the GO enrichment analysis and the top 20 outcomes from the KEGG pathway enrichment analysis were subsequently visualized.

### 2.5 Molecular Docking

The InChIKey representations of the chosen core compounds from PEE were sourced from the PubChem database. Mol2 files were generated, followed by energy minimization using Chem3D software (CambridgeSoft, Cambridge, MA, USA). The three‐dimensional structures of the key protein targets were retrieved from the Protein Data Bank (PDB) database (http://www.rcsb.org/). Preparation of the proteins included the removal of ligands and water molecules, as well as the addition of polar hydrogen atoms. Molecular docking simulations were performed utilizing AutoDock Vina, and the outcomes were visualized and analyzed with PyMOL software (https://pymol.org). Binding affinity values are presented in kJ/mol, where scores below −5 signify moderate binding activity, and scores below −7 indicate strong binding activity.

### 2.6 Animals and Experimental Protocol

#### 2.6.1 Acute Toxicity Test

Twenty Sprague–Dawley (SD) rats were housed in the standardized animal facility of the Wanbei Coal Electric Group General Hospital and allowed to acclimate for 5 days with unrestricted access to food and water. The rats were randomly assigned to two groups: a control group and a treatment group, each comprising five males and five females. The treatment group received a maximum intraperitoneal gavage dose of 1000 mg/kg, whereas the control group was administered an equivalent volume of normal saline (Kennedy et al. 1986). Toxic reactions were meticulously monitored for 48 h following administration. In instances where death occurred within 14 days post‐administration, an immediate necropsy was performed to observe macroscopic pathological changes in major organs, including the brain, heart, spleen, liver, kidneys, lungs, stomach, and intestines. After the 14‐day period, the surviving animals were euthanized using isoflurane anesthesia, and blood samples were collected for hematological and biochemical analyses in accordance with relevant literature (Arpornchayanon et al. 2022). The primary organs (heart, liver, spleen, lungs, kidneys) were excised, thoroughly rinsed with pre‐cooled saline, weighed, and then fixed in 4% paraformaldehyde for 48 h to prepare for subsequent histopathological examination.

#### 2.6.2 Construction of Pneumonia Model Mice

Forty Sprague–Dawley (SD) rats were housed in a standardized animal facility at the Wanbei Coal Electric Group General Hospital (Animal Ethics Approval Number SCXK2023‐030) and given unrestricted access to food for 1 week to acclimate to their surroundings. All experimental procedures adhered to national ethical guidelines for animal research. The rats were randomly assigned to five groups, with eight rats in each group: a control group (C), a model group receiving lipopolysaccharide (LPS) at 5 mg/kg, a positive control group treated with Dexamethasone at 5 mg/kg, and three experimental groups administered Ganodermatriol at low (25 mg/kg), medium (50 mg/kg), and high (100 mg/kg) doses.

To establish a pneumonia model, rats received an intratracheal instillation of LPS at a dosage of 5 mg/kg. The LPS was prepared by dissolving it in sterile saline to achieve a concentration of 2 mg/mL, protected from light, and used within 1 week as per previous studies (Burkard et al. 2023; Klein et al. 2023). Prior to establishing the pneumonia model, all rats were fasted for 12 h, although water remained available. The positive control and experimental groups were administered their respective treatments via gavage at a volume of 5 mL/kg, while the control and model groups received an equivalent volume of saline through gavage. 12 h after the initial modeling, the same dosages were administered again orally. Treatments were continued once daily for 20 consecutive days following the modeling procedure. Throughout the treatment period, rats were monitored and weighed regularly. At the end of the study, the rats were euthanized, and relevant samples were collected for analysis based on the experimental indicators. Additionally, food was withheld for 12 h before euthanasia, although water was accessible.

#### 2.6.3 Daily Monitoring and Sample Collection

Daily monitoring was conducted for each group, recording alterations in fur quality of rats, mental condition, behavioral patterns, food intake, and fecal output. On the 21st day, the rats were weighed and then subjected to overnight fasting while having unrestricted access to water. The dosage of sodium pentobarbital was determined based on each rat's body weight, and anesthesia was administered via intraperitoneal injection of 1% sodium pentobarbital (10 mg/mL). Both lungs were meticulously excised and rinsed with cold saline to remove any blood residues. After drying with filter paper, the wet lung weight of each rat was measured, and the lung coefficient was calculated using the following formula:

(1) $$\text{Lung Coefficient}=\mathrm{Wet}\;\text{Lung Weight}\;\left(g\right)/\text{Body Weight}\;\left(g\right)\times 100\%$$

#### 2.6.4 Hematoxylin and Eosin and Masson Staining

After being fixed in a 4% paraformaldehyde/PBS (v/v) solution for 48 h, the lung tissues were paraffin‐embedded and sliced into sections with a thickness of 5 μm. The sections were stained with hematoxylin and eosin (H&E) as well as Masson's trichrome, following established standard protocols (Zhang et al. 2023). Evaluation of alveolitis and pulmonary interstitial fibrosis was conducted based on the H&E‐stained sections, utilizing the scoring methodologies outlined by Ashcroft et al. (1988); Szapiel et al. (1979).

#### 2.6.5 Elisa Test

A lung tissue homogenate was prepared at a concentration of 10% (w/v) and subsequently centrifuged at 4000 rpm for 10 min at 4°C. The resulting supernatant was then collected for biochemical assays. The concentrations of TNF‐α, IL‐6, and IL‐1β were quantified utilizing commercial ELISA kits, adhering closely to the manufacturer's instructions. Absorbance measurements were taken at a wavelength of 450 nm using a microplate reader (Thermo Fisher Scientific, Waltham, MA, USA). Cytokine levels were expressed in pg/mL.

#### 2.6.6 Western Blotting

An equal quantity of protein (50 μg) was introduced into each well of a 7.5% sodium dodecyl sulfate‐polyacrylamide gel. After electrophoresis, the proteins were transferred onto a polyvinylidene fluoride (PVDF) membrane. The membrane was initially blocked with non‐fat milk and subsequently incubated overnight at 4°C with primary antibodies. Western blot analysis was performed to evaluate the expression levels of NF‐κB, p‐NF‐κB P65, IκBα, p38, p‐Ikkα/β, p‐p38, Erk1/2, p‐Erk1/2, JNK1/2/3, p‐JNK1/2/3, and GAPDH. Following this, the membrane was incubated with a secondary HRP‐conjugated anti‐rabbit antibody. Chemiluminescent signals were detected using an enhanced substrate, and densitometric analysis was carried out with ImageJ software (Bethesda, MD, USA).

### 2.7 Statistical Analysis

Experimental data were analyzed using GraphPad Prism version 10.3.5 software, and the results are presented as means ± standard deviation. For datasets that adhered to a normal distribution, a One‐Way ANOVA was employed. In cases where the data deviated from normality, the Kruskal‐Wallis test was used. All data were obtained from at least three independent experiments, and statistical significance was determined at *p* < 0.05.

## 3 Results

### 3.1 Phytochemical Characterization of GLA

**FIGURE 1:**
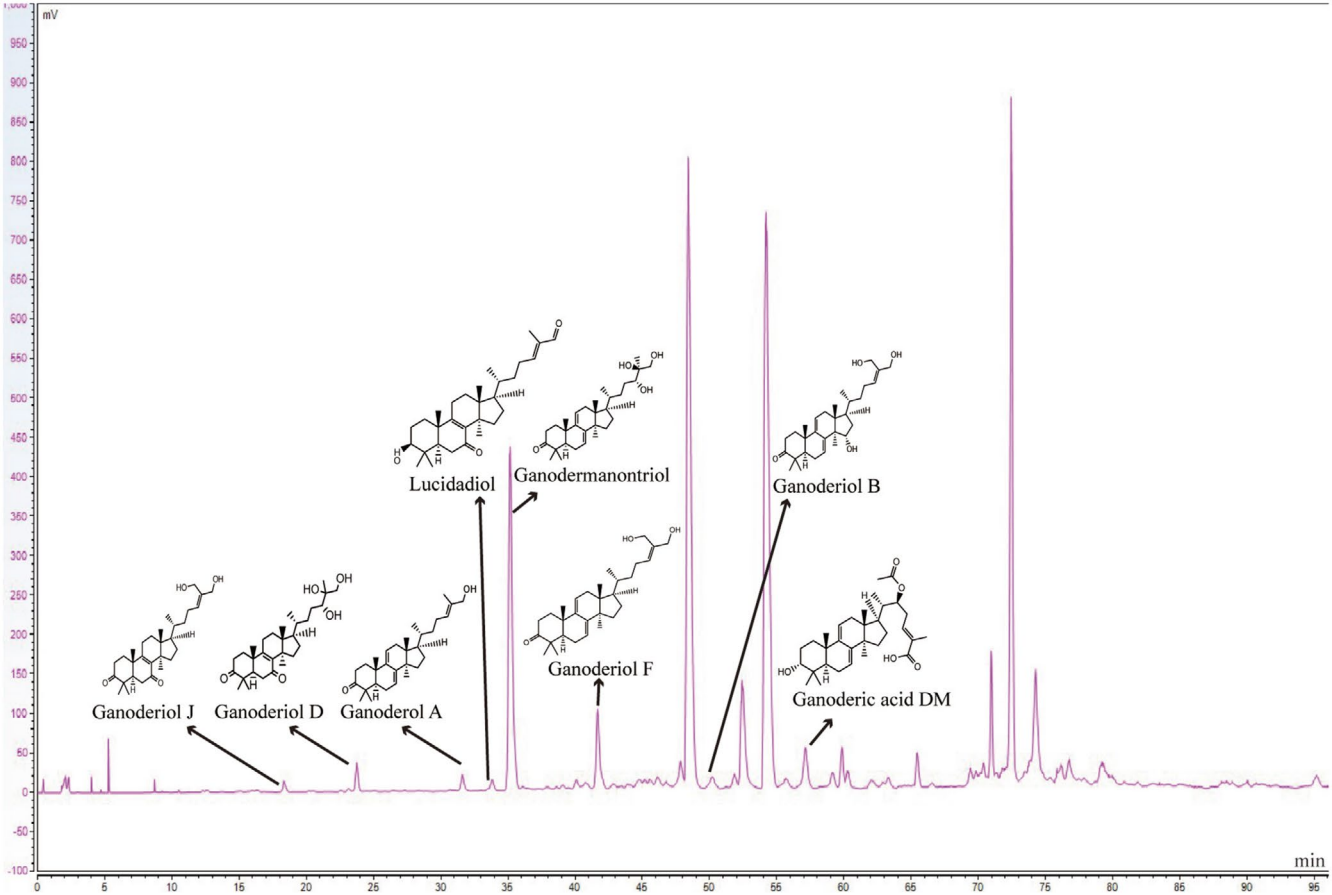
Fingerprint analysis of triterpenes from the ethanol extract of *G. lucidum* .

The quantitative analysis revealed that the total triterpenoid content in GLA was 36.14 ± 0.98 mg of oleanolic acid equivalents per gram, accounting for 36.14% ± 0.98% of the extract. The HPLC chromatogram of GLA identified eight triterpenoids by comparing their retention times and diode‐array detector (DAD) spectra with those of the corresponding reference standards (Figure 1, Table S2). For quantification, the total ion chromatograms (TIC) are illustrated in Figures S1–S3. Table 1 presents the linear regression data, correlation coefficients (R values), and the quantified concentrations of four triterpenoids. Notably, ganodermanontriol was determined to be the most abundant triterpenoid in GLA, with a concentration of 110,345.12 ± 0.41 mg/kg.

**TABLE 1:** The content of 4 substances in GLA by LC–MS.

Compounds	Regression equation	R	Contents(mg/kg)
Ganoderiol D	*y* = 195.11x + 7888.30	0.9975	2008.23 ± 0.23
Ganodermanontriol	*y* = 3.3973x + 71.225	0.9991	110345.12 ± 0.41
Ganoderiol F	*y* = 12.067x + 528.11	0.9978	2652.52 ± 0.24
Ganoderic acid DM	*y* = 43.58x + 1337.90	0.9976	1643.81 ± 0.38

*Note:* The reported values are expressed as mean ± standard deviation (*n* = 3) of triplicate measurements.

### 3.2 Network Pharmacology Analysis

#### 3.2.1 Network Pharmacology‐Based Analysis

**FIGURE 2:**
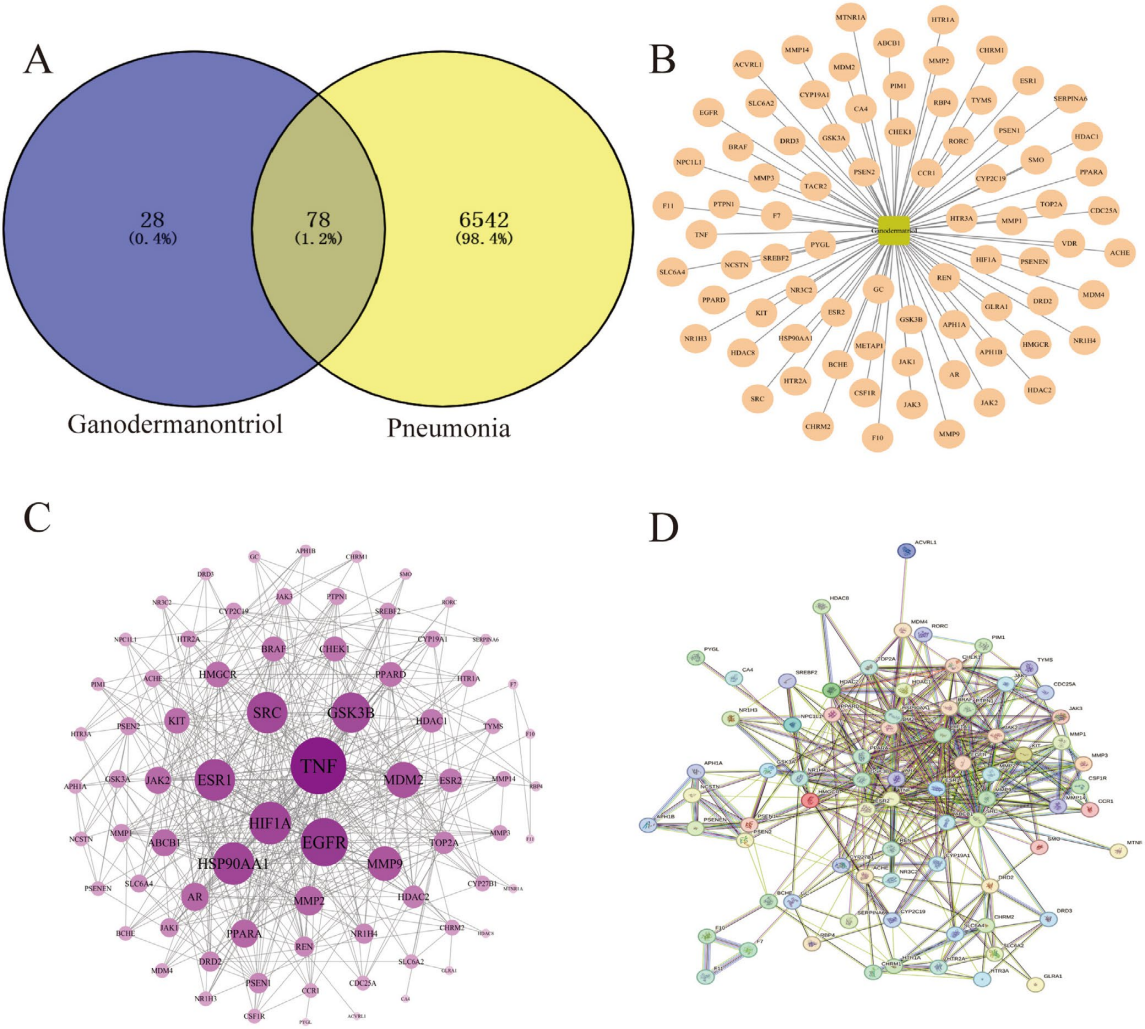
(A) Venn diagram of active ingredients and disease targets, (B) Ganodermanontriol active ingredient‐target network, (C) Protein–protein interaction (PPI) network of key targets, (D) STRING database PPI network depicting interactions between different proteins.

To identify disease‐associated targets, the keyword “pneumonia” was used to search relevant databases, consolidating the collected targets and removing duplicates, resulting in 6542 unique targets for pneumonia. Subsequently, the drug‐related targets of Ganodermanontriol were identified using Swiss Target Prediction and confirmed through the UniProt database, resulting in the identification of 106 active targets of Ganodermanontriol. A comparative analysis between the disease‐related and drug‐related targets was performed using Venny 2.1.0, which produced a Venn diagram illustrating 78 common targets (Figure 2A). Additionally, network analysis revealed the interaction network between the drug and disease targets (Figure 2B), highlighting intricate relationships and indicating that the drug may exert its therapeutic effects through multiple pathways.

#### 3.2.2 Analysis of Protein–Protein Interaction (PPI) Network

The 78 overlapping targets were subjected to protein–protein interaction (PPI) analysis using the STRING database. The resulting interaction data were visualized in Cytoscape to generate a PPI network, with node coloration intensity reflecting interaction scores—darker nodes denoting stronger interactions. As shown in Figure 2C, TNF displayed the deepest coloration, suggesting its central role in the plant's anti‐inflammatory mechanism. Core targets were further identified via the Maximal Clique Centrality (MCC) algorithm, and a refined core PPI network was constructed (Figure 2D).

#### 3.2.3 GO Analysis and KEGG Pathway Enrichment Analysis

**FIGURE 3:**
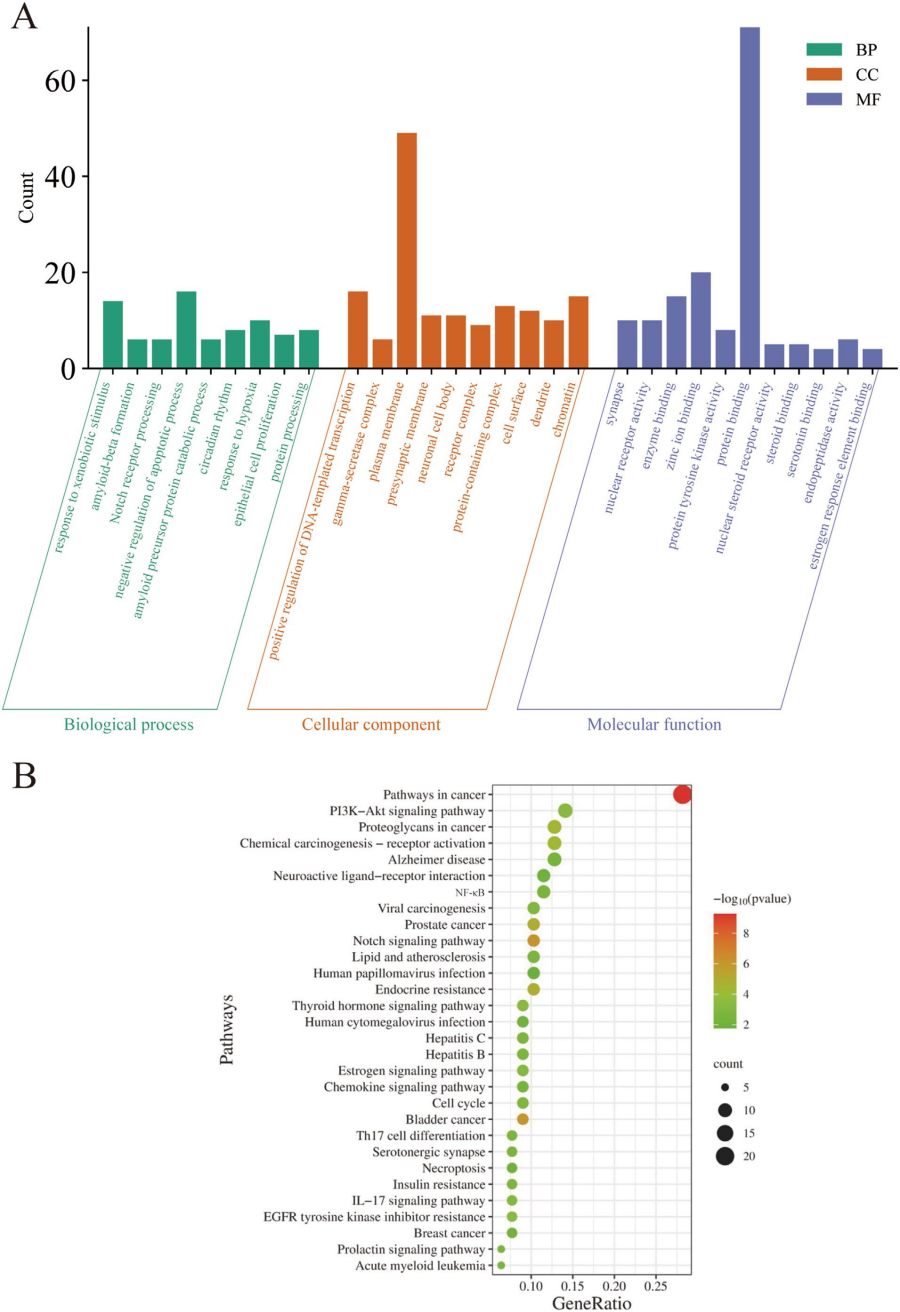
(A) GO functional enrichment analysis; (B) KEGG functional enrichment analysis.

Gene Ontology (GO) functional enrichment analysis of the targets within the PPI network was carried out using Metascape. The analysis identified significant enrichment in 10 Cellular Component (CC), 11 Molecular Function (MF), and 9 Biological Process (BP) categories (*p* < 0.05). The most prominently enriched biological processes included response to metabolic stimuli, negative regulation of apoptosis, the Notch signaling pathway, and embryonic developmental rhythms, all closely related to cellular metabolism and signal transduction. In terms of cellular components, the top enriched categories were presynaptic membrane, protein complex, cytoplasm, and chromatin, indicating that these targets primarily operate within specific cellular structures and regions. Regarding molecular functions, nuclear receptor activity showed significant enrichment, alongside enzyme binding and transcription factor activity, underscoring their vital roles in signal transduction and gene regulation. These GO enrichment findings suggested that the targets are involved in various biological processes, particularly in cell signaling, organelle structural maintenance, and gene expression regulation (Figure 3A).

Pathway enrichment analysis of the targets was conducted using the KEGG database, uncovering several significantly enriched pathways (*p* < 0.05). The top 20 most significantly enriched pathways are depicted in Figure 3B. The analysis revealed that “Pathways in cancer” was the most significantly enriched pathway. Additionally, the PI3K–Akt signaling pathway, proteoglycans in cancer, chemical carcinogenesis—receptor activation, and Alzheimer's disease pathways were also notably enriched. These results suggest that many of the identified pathways are associated with cancer progression and immune regulation. Subsequently, ganodermanontriol and the core targets were selected for molecular docking studies.

#### 3.2.4 Molecular Docking

**FIGURE 4:**
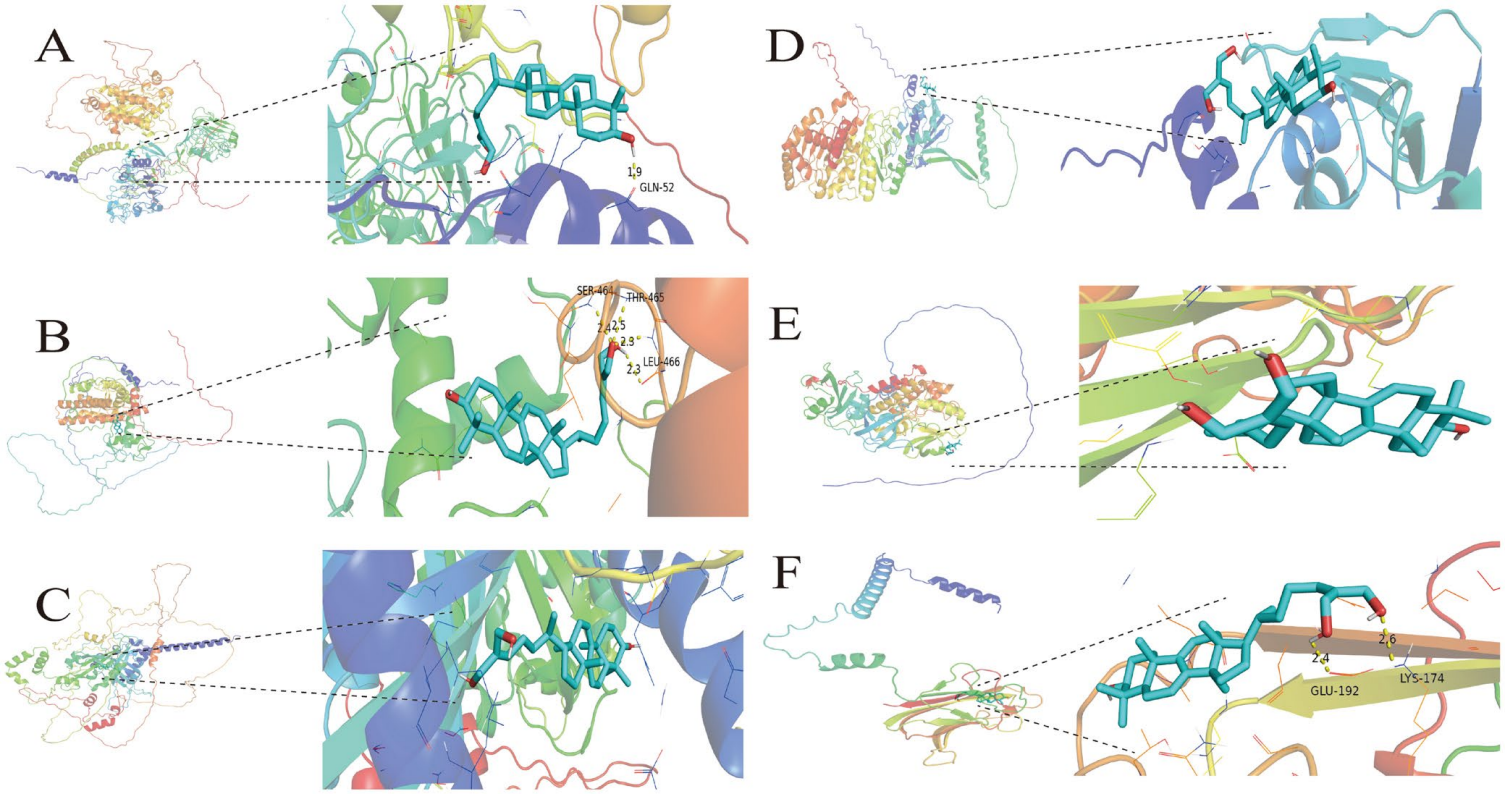
The binding affinities of ganodermanontriol for EGFR, TNF, ESR1, HIF1A, SRC, and HSP90AA1(A‐F).

Typically, a binding energy below‐5 kcal/mol between a ligand and its target protein signifies a stable interaction, with more negative values indicating stronger binding affinities. The molecular docking analysis revealed that all observed binding energies were below‐5 kcal/mol, as shown in Table 2. As depicted in Figure 4, ganodermanontriol demonstrated strong binding affinities with EGFR, TNF, ESR1, HIF1A, SRC, and HSP90AA1, suggesting robust binding stability.

**TABLE 2:** Binding energy.

Targets	Binding energy (kcal/mol)
EGFR	−5.4
ESR1	−6.8
HIF1A	−6.6
HSP90AA1	−6.1
TNF	−6.9
SRC	−5.9

### 3.3 In Vivo Acute Toxicity of Ganodermanontriol

**FIGURE 5:**
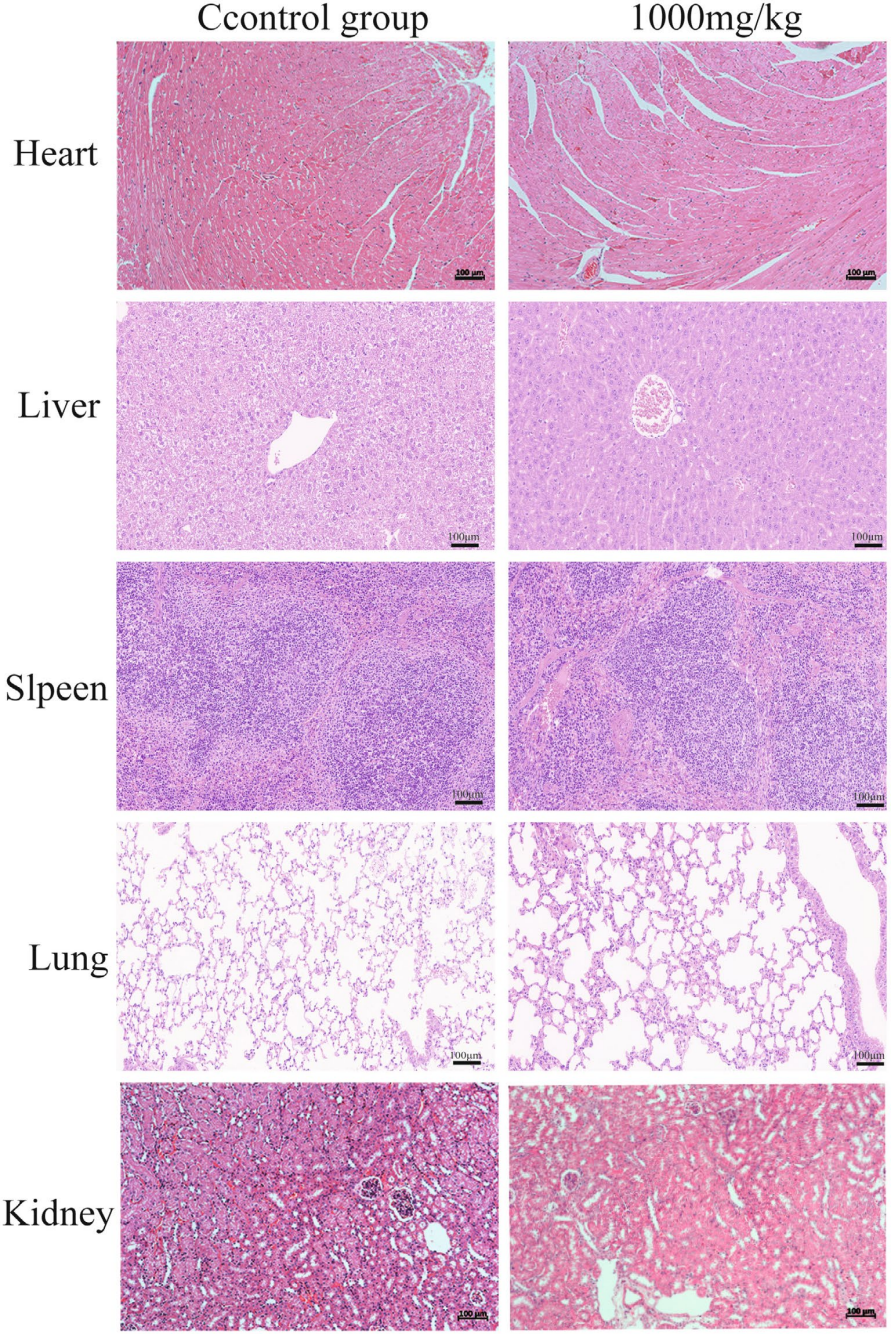
Effect of the Ganodermanontriol on the microstructures of organs in rats (Scale bar = 100 μm).

In acute toxicity assessments, 30 min after oral gavage administration, both male and female rats displayed increased water consumption, reduced activity levels, and a tendency to curl into a ball. These behaviors gradually diminished after 1 h, and by 4 h post‐administration, the rats had returned to their normal state, showing no noticeable differences compared to the control group. No additional toxic reactions were observed during 48 h of close monitoring. Over the subsequent 14 days, there were no fatalities, and all rats maintained normal drinking, feeding, and activity behaviors. Upon euthanasia, no observable pathological changes were detected in the heart, liver, spleen, lungs, and kidneys, either macroscopically or microscopically (Figure 5).

### 3.4 Anti‐Pneumonia of Ganodermanontriol

#### 3.4.1 Effect of Ganodermanontriol on Pathological Morphology of Pneumonia Rats

**FIGURE 6:**
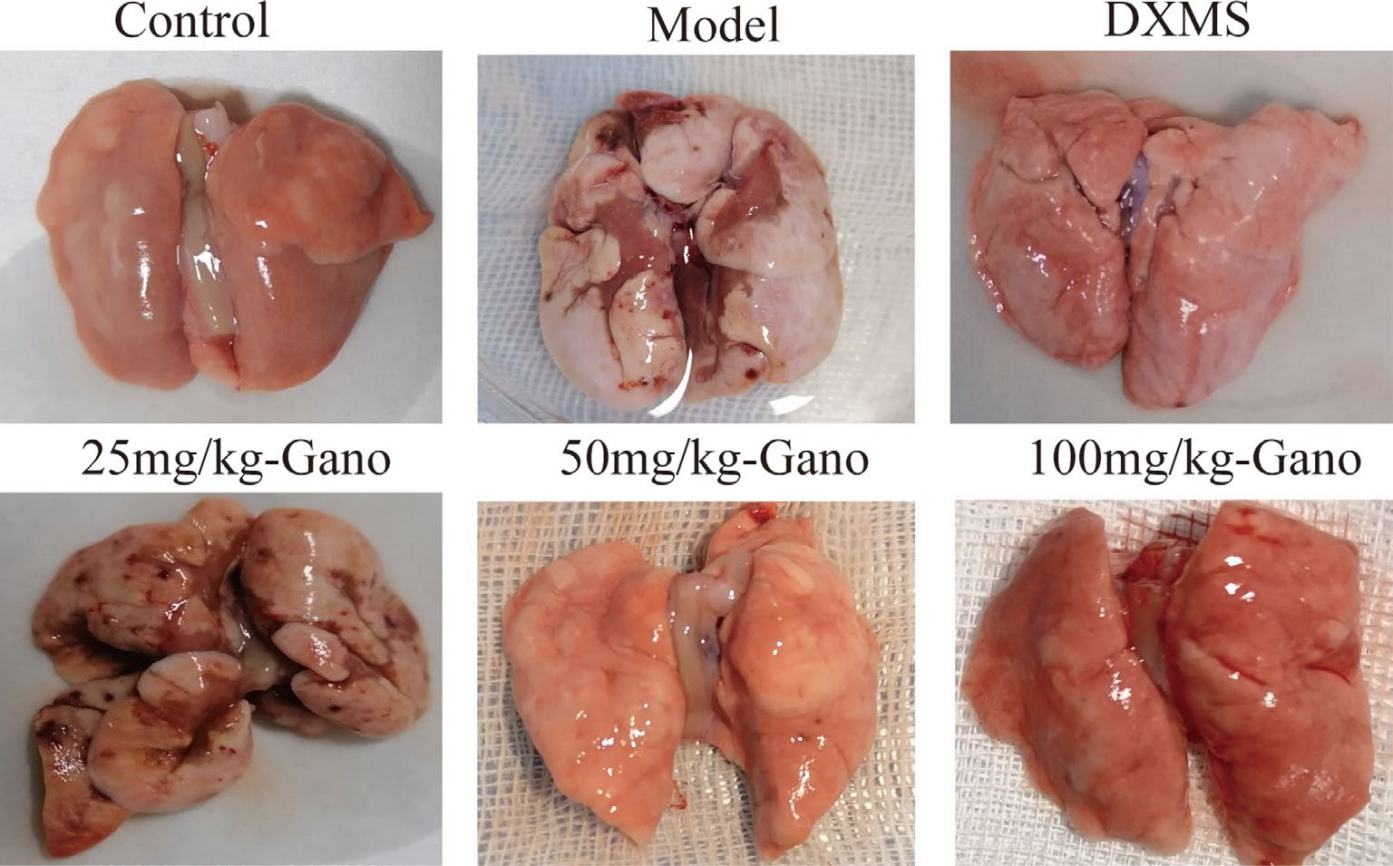
Gross morphological changes in rat lungs from different treatment groups. DXMS, dexamethasone; Gano, ganodermanontriol.

The gross lung morphology of rats across different groups, including the control group, model group, dexamethasone‐treated group (DXMS), and three ganodermanontriol dosage groups (25 mg/kg, 50 mg/kg, and 100 mg/kg), was examined. As shown in Figure 6, the control group displayed lungs with intact structures and a healthy pink color. In contrast, the model group exhibited clear congestion, edema, and pathological changes associated with pneumonia. The dexamethasone‐treated group showed significant improvement in lung inflammation, with reduced signs of edema and lesion formation. Among the ganodermanontriol‐treated groups, the 25 mg/kg dosage still presented considerable inflammation; however, inflammation progressively diminished in the 50 mg/kg and 100 mg/kg groups. Notably, the 100 mg/kg group's lungs closely resembled those of the control group. Analysis of the lung organ index revealed that ganodermanontriol effectively reversed lipopolysaccharide (LPS)‐induced pulmonary edema in a dose‐dependent manner (Figure 9A).

**FIGURE 7:**
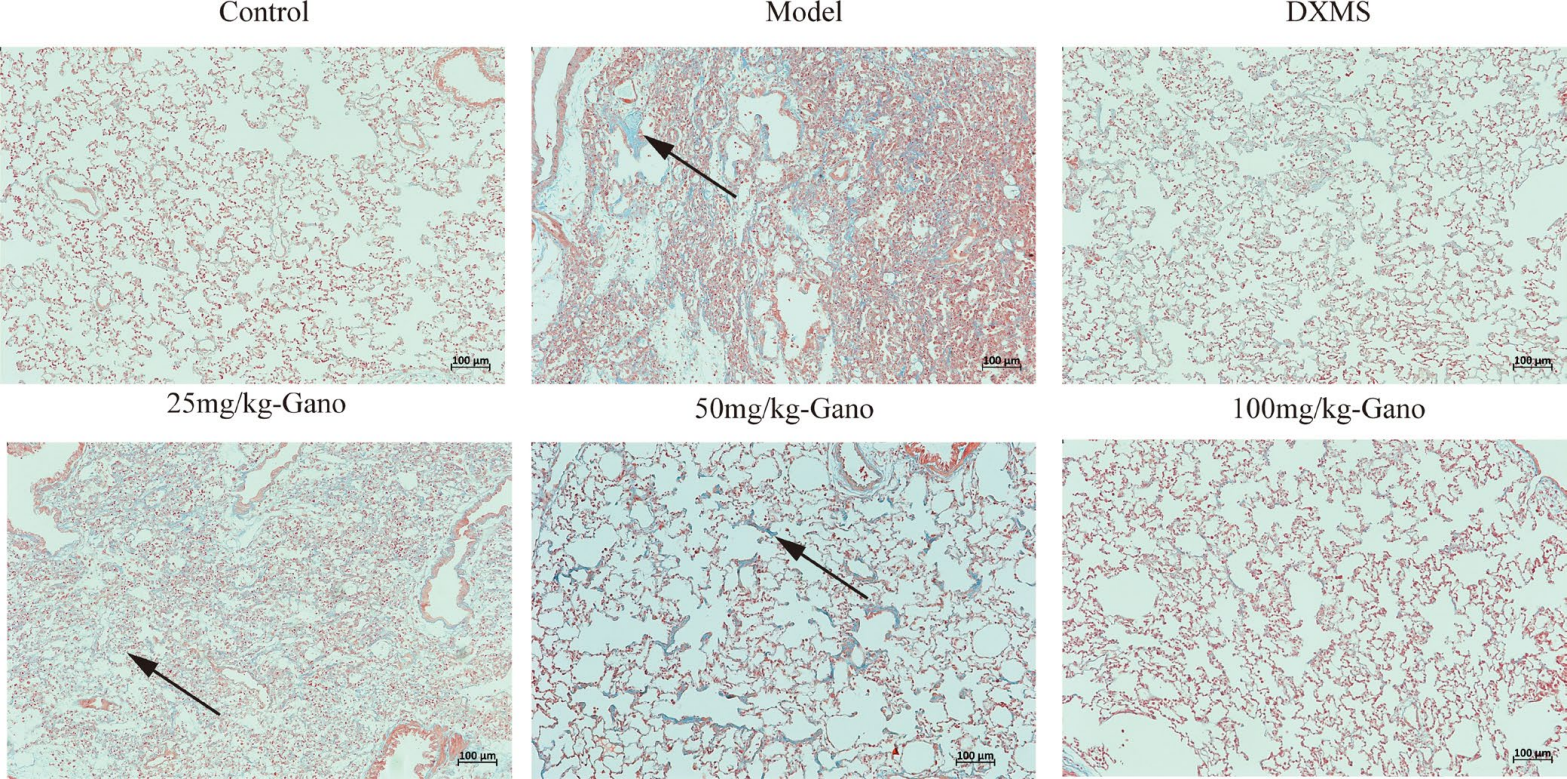
Histopathological examination of rat lung tissues following different treatments. DXMS, dexamethasone; Gano, ganodermanontriol. Scale bar = 100 μm.

Histological examinations of lung tissues revealed that, compared to the control group, rats in the model group exhibited diffuse thickening of the alveolar septa and uneven destruction of the alveolar walls (Figure 7). There was an increase in alveolar–capillary membrane thickness, accompanied by significant neutrophil infiltration into the alveoli and septa. Additionally, alveolar and interstitial edema, hyaline membrane formation, extensive inflammatory cell infiltration, and scattered hemorrhages were evident. In the low‐dose ganodermanontriol treatment group, some improvement was observed; however, certain lung lobes remained notably damaged, with uneven lesions and persistent inflammatory cell infiltration, including irregular alveolar septal thickening and hemorrhagic spots. In the medium‐ and high‐dose ganodermanontriol groups, alveolar structures showed marked improvement, with substantial alleviation of interstitial thickening and edema. The extent of inflammatory cell infiltration was significantly reduced, and hemorrhagic occurrences were fewer. In the positive control group, there was a significant improvement in the thickening of alveolar tissues, with alveolar wall structures largely intact and minimal inflammatory cell infiltration. Edema in both the interstitium and alveoli was also reduced. According to the Szapiel scoring system (Figure 9B), alveolitis was significantly elevated in the model group compared to the control group (*p* < 0.001). The HE scores in the low‐, medium‐, and high‐dose ganodermanontriol groups progressively decreased compared to the model group (*p* < 0.05, *p* < 0.01, *p* < 0.001), indicating that ganodermanontriol significantly mitigated LPS‐induced alveolitis in rats in a dose‐dependent manner.

**FIGURE 8:**
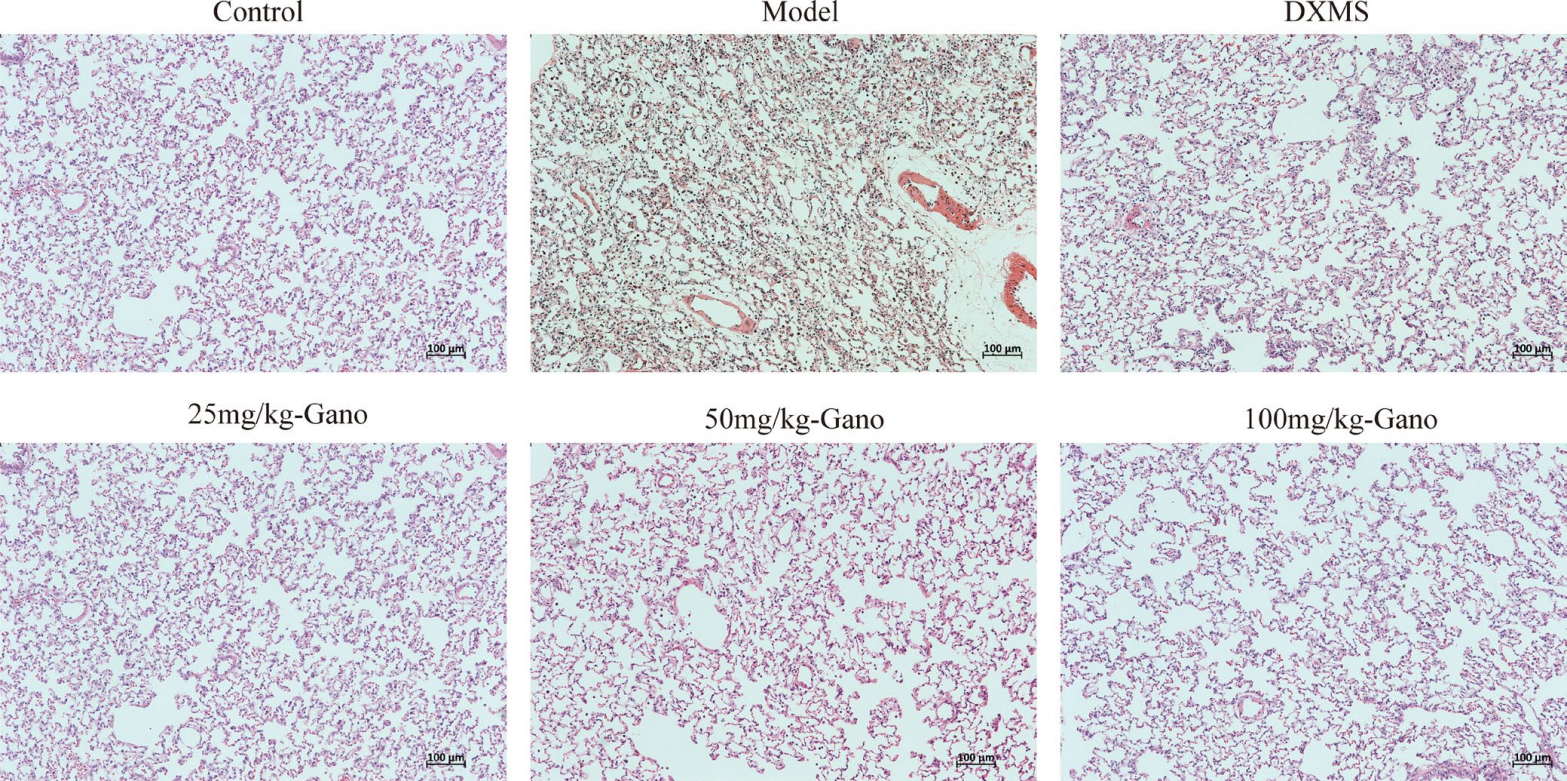
Masson staining of rat lung tissues following different treatments. DXMS, dexamethasone; Gano, ganodermanontriol. Scale bar = 100 μm.

**FIGURE 9:**
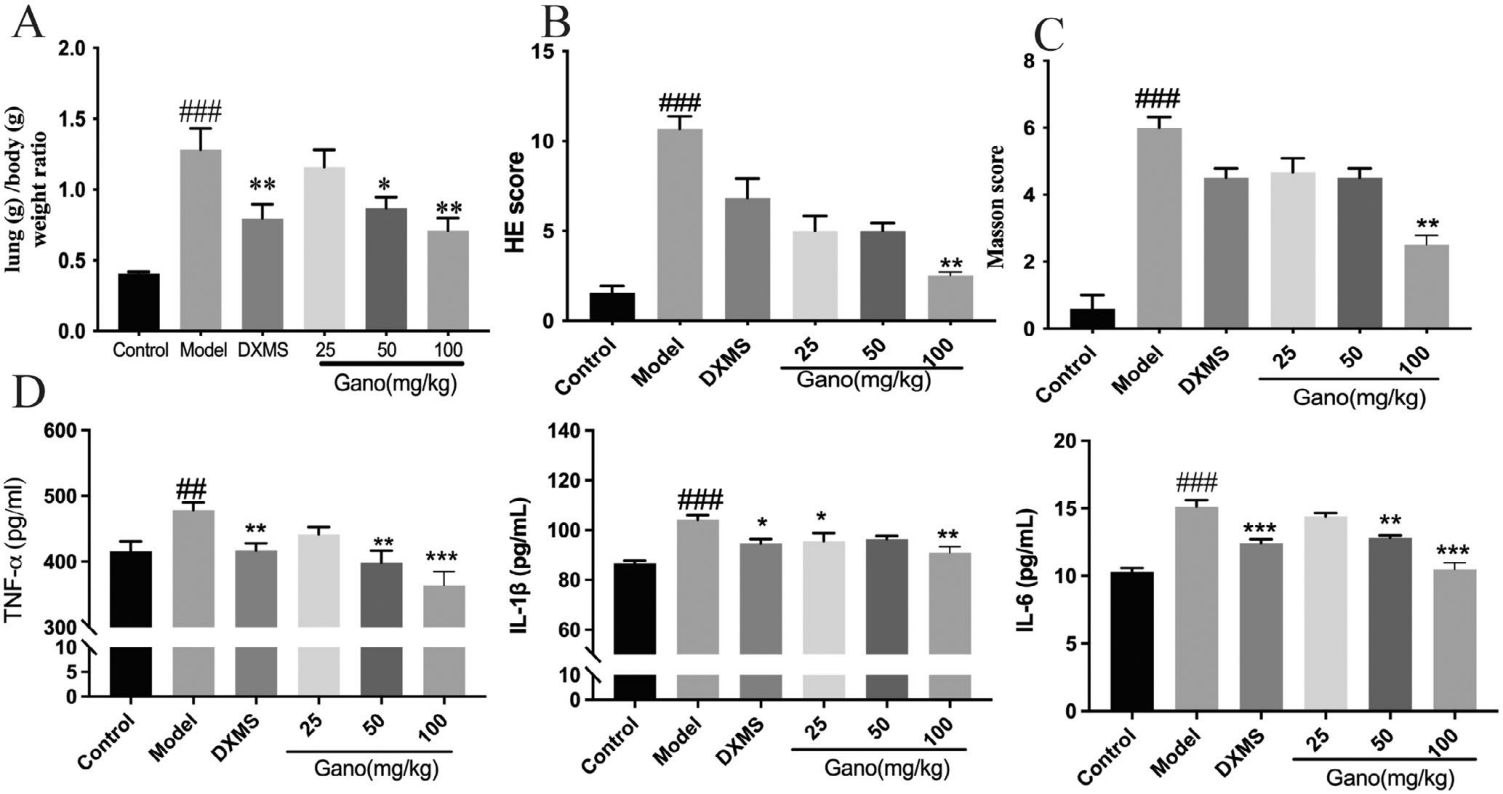
Quantitative Evaluation of Lung Injury and Fibrosis in Different Treatment Groups. (A) Lung wet weight/body weight ratio; (B) HE scores representing alveolitis severity; (C) Masson score indicating collagen deposition in lung tissue; (D) Ganodermanontriol reduced inflammatory factors in the lung of rats. DXMS, Dexamethasone; Gano, ganodermanontriol. Compared to the control group, ^###^ *p* < 0.001, ^##^ *p* < 0.01; compared to the model group, *p < 0.05, **p < 0.01, ***p < 0.001.

Upon histopathological analysis with Masson's trichrome staining (Figures 8, 9C), lung tissues in the model group demonstrated extensive deposition of collagen fibers, evidenced by prominent blue staining, which exhibited markedly increased density relative to control groups. In contrast, the ganodermanontriol‐treated groups (low, medium, and high doses) demonstrated a dose‐dependent reduction in collagen fiber deposition, highlighting the compound's antifibrotic effects (Figures 6, 7, 8).

#### 3.4.2 Effect of Ganodermanontriol on Inflammatory Factors in Rat Lung Tissue

Tumor necrosis factor‐alpha (TNF‐α), interleukin‐1 beta (IL‐1β), and interleukin‐6 (IL‐6) are key pro‐inflammatory cytokines. Enzyme‐linked immunosorbent assay (ELISA) results, as depicted in Figure 9D, indicated that the low‐dose ganodermanontriol group exhibited a reduction in IL‐1β, TNF‐α, and IL‐6 levels compared to the model group; however, these changes were not statistically significant (*p* > 0.05). In contrast, the high‐dose ganodermanontriol group demonstrated a significant downregulation of all inflammatory cytokines, markedly lowering their expression levels. Overall, ganodermanontriol displayed excellent anti‐inflammatory activity and protective effects on lung tissue at high doses, with its efficacy demonstrating a dose‐dependent relationship.

#### 3.4.3 Effects of Ganodermanontriol on the Expression of TNF/NF‐κB/MAPK Related Signaling Pathways in Rats

**FIGURE 10:**
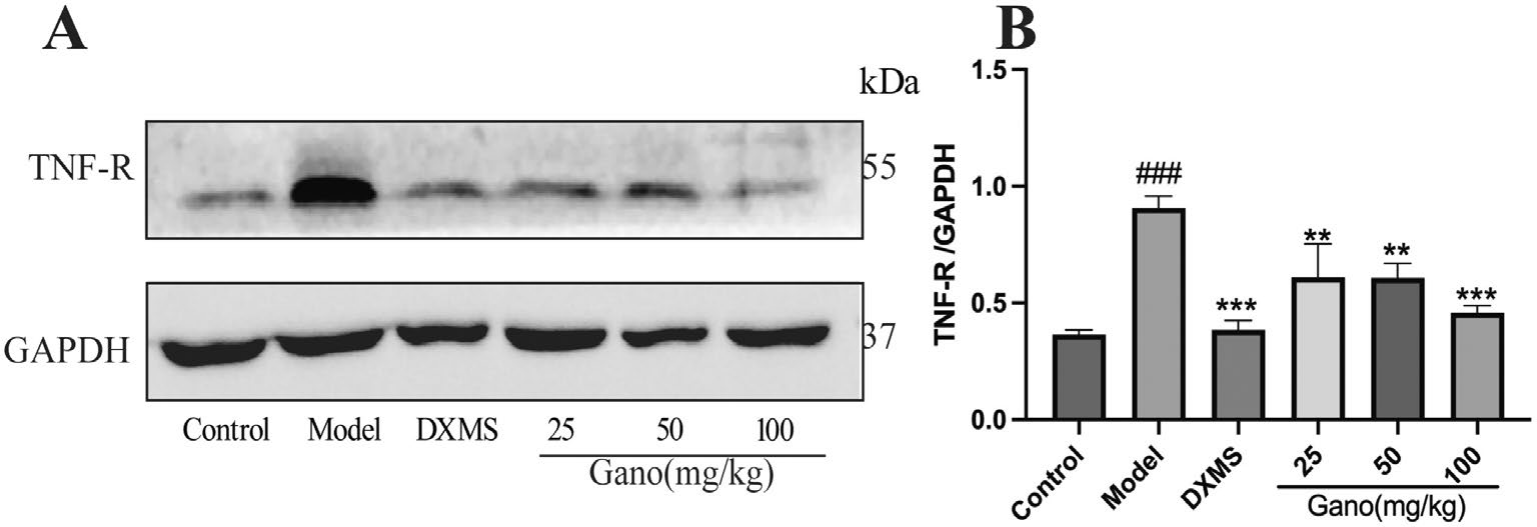
TNF‐R expression in rat lung tissue following different treatments. (A) Western blot analysis of TNF‐R protein levels; (B) Quantitative analysis of TNF‐R expression relative to GAPDH; DXMS, Dexamethasone; Gano, ganodermanontriol. Compared to the control group, ^###^ *p* < 0.001; compared to the model group, **p < 0.01, ***p < 0.001.

Tumor necrosis factor receptor (TNF‐R) is a key pro‐inflammatory marker. Treatment with dexamethasone and various doses of ganodermanontriol significantly reduced TNF‐R expression compared to the model group (*p* < 0.01 or *p* < 0.001) (Figure 10). Notably, in the 50 mg/kg and 100 mg/kg dosage groups, TNF‐R expression levels decreased markedly, approaching those observed in the control group. This indicates that ganodermanontriol effectively inhibits TNF‐R expression in a dose‐dependent manner, thereby attenuating the inflammatory response.

**FIGURE 11:**
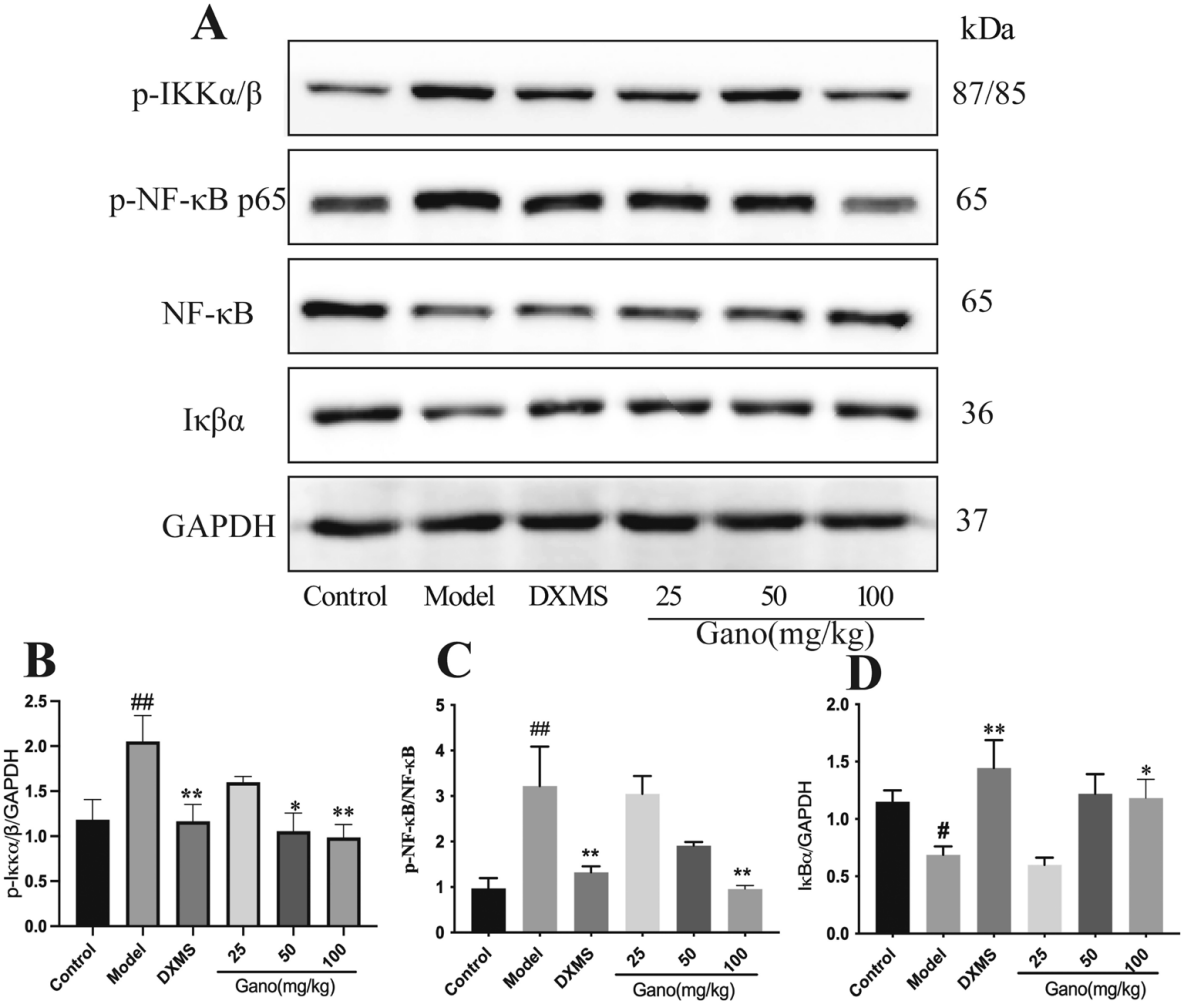
Western blot analysis of NF‐κB pathway activation in rat lung tissues. (A) Representative Western blots of phosphorylated IKKα/β (p‐IKKα/β), phosphorylated NF‐κB p65 (p‐NF‐κB p65), total NF‐κB, and IκBα; (B–D) Quantification of p‐IKKα/β, p‐NF‐κB p65, and IκBα protein levels relative to GAPDH. DXMS, Dexamethasone; Gano, ganodermanontriol. Compared to the control group, ^#^ *p* < 0.05, ^##^ *p* < 0.01; compared to the model group, *p < 0.05, **p < 0.01.

Histological analysis revealed that, compared to the control group, rats in the model group exhibited significantly elevated levels of phosphorylated IKKα/β (p‐IKKα/β) and phosphorylated NF‐κB p65 (p‐NF‐κB p65), alongside markedly decreased expression of IκBα (Figure 11). Treatment with various doses of ganodermanontriol significantly reduced the levels of p‐IKKα/β and p‐NF‐κB p65 compared to the model group (*p* < 0.05 or *p* < 0.01), demonstrating a dose‐dependent effect. Furthermore, in the 100 mg/kg dosage group, IκBα expression was significantly higher than in the model group (*p* < 0.05), suggesting that high‐dose ganodermanontriol effectively inhibits NF‐κB pathway activation, thereby mitigating the inflammatory response.

**FIGURE 12:**
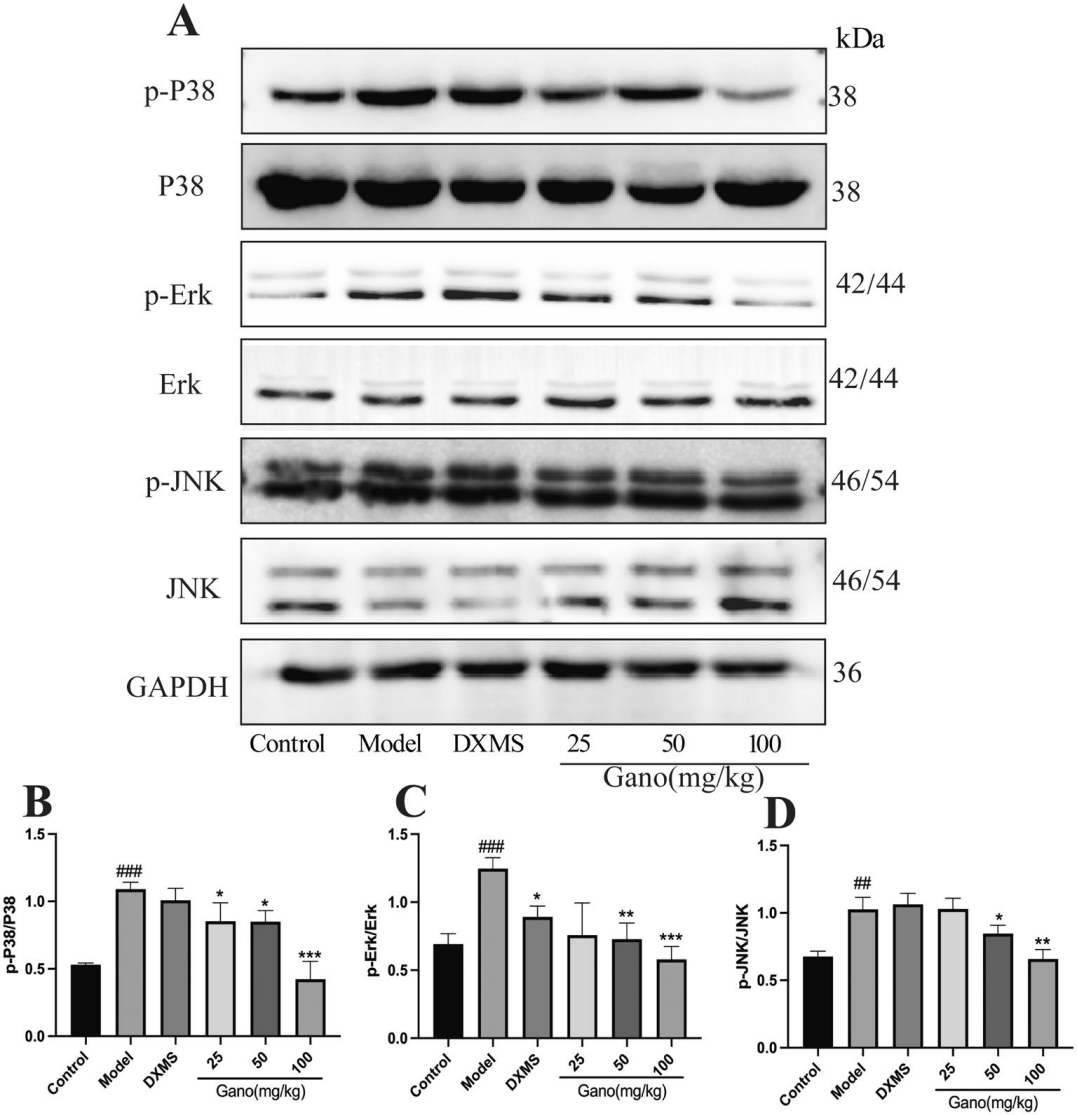
Effects of ganodermanontriol on MAPK pathway activation in rat lung tissues. (A) Representative Western blots showing phosphorylated P38 (p‐P38), total P38, phosphorylated Erk (p‐Erk), total Erk, phosphorylated JNK (p‐JNK), total JNK, and GAPDH; (B–D) Quantification of p‐P38/P38, p‐Erk/Erk, and p‐JNK/JNK protein levels. DXMS, Dexamethasone; Gano, ganodermanontriol. Compared to the control group, ^##^ *p* < 0.01, ^###^ *p* < 0.001; compared to the model group, *p < 0.05, **p < 0.01, ***p < 0.001.

Compared to the control group, the model group showed significantly elevated phosphorylation levels of p‐P38, p‐ERK, and p‐JNK, indicating activation of the MAPK signaling pathway in acute lung injury (Figure 12). Treatment with dexamethasone (DXMS) and various doses of ganodermanontriol significantly reduced the phosphorylation levels of p‐P38, p‐ERK, and p‐JNK in the 50 mg/kg and 100 mg/kg groups (*p* < 0.05, *p* < 0.01), demonstrating a dose‐dependent inhibitory effect on the MAPK signaling pathway.

## 4 Discussion

Ganodermanontriol is a significant metabolite of *G. lucidum* , demonstrating pharmacological activities such as anti‐inflammatory, antimicrobial, and antitumor effects (Hu et al. 2024, 2020; Zhang et al. 2024). In this study, eight active compounds in *G. lucidum* were identified using HPLC and LC–MS methods. These compounds were further analyzed through network pharmacology and molecular docking to predict potential targets and pathways underlying *G. lucidum* 's anti‐pneumonia effects. The results indicated that the ethanol extract of *G. lucidum* contains eight tetracyclic triterpenoids, with ganodermanontriol being the most abundant.

Based on the HPLC and LC–MS findings, we focused on the most abundant component, ganodermanontriol, for network pharmacology and molecular docking studies. These analyses identified 106 potential targets. Enrichment analysis of these targets yielded 30 Gene Ontology (GO) functional terms and 29 Kyoto Encyclopedia of Genes and Genomes (KEGG) pathways. Critical targets such as TNF, EGFR, ESR1, HIF1A, HSP90AA1, and SRC were involved in pathways including chemical carcinogenesis, PI3K‐Akt signaling, proteoglycans in cancer, receptor activation in chemical carcinogenesis, and immunomodulation. TNF, also known as TNF‐α (Huyghe et al. 2023), is predominantly secreted by neutrophils, activated lymphocytes, and macrophages. It can induce apoptosis in certain tumor cell lines and primarily participates in systemic inflammatory responses, cell death, and acute‐phase reactions (Pan et al. 2022). TNF‐α interacts with two types of receptors: TNFR1, which is expressed in most tissues and contains a death domain that recruits the adaptor protein TRADD (TNFR1‐associated death domain protein). Upon TNF‐α binding to either soluble or membrane‐bound TNF through TNFR1, complex I is assembled, comprising key molecules such as TRADD, RIPK1, TRAF2, and the LUBAC complex (Jang et al. 2021). Through ubiquitination, LUBAC adds linear ubiquitin chains, stabilizing complex I and amplifying the signal. This process activates two crucial signaling complexes: the TAK1 complex, which initiates the MAPK signaling cascade by activating JNK, p38, and AP1 transcription factors; and the IKK complex, which activates the NF‐κB pathway (Chen and Goeddel 2002). Molecular docking studies revealed that ganodermanontriol's binding energies with core target proteins (TNF, EGFR, ESR1, HIF1A, HSP90AA1, SRC) were all below −5.0 kcal/mol, indicating stable binding activities primarily mediated by hydrogen bonds (Li and Li 2024). Furthermore, binding sites were identified, confirming that ganodermanontriol exhibits strong binding characteristics with these targets. Consequently, the TNF/NF‐κB/MAPKs signaling pathway was selected to investigate the potential mechanisms by which ganodermanontriol treats pneumonia.

Prior to in vivo experiments, acute toxicity tests demonstrated that ganodermanontriol showed no toxicity in Sprague–Dawley (SD) rats at a dose of 1000 mg/kg, establishing a safe dosing foundation for subsequent activity studies. Comprehensive safety evaluations should be conducted in future research (Deng et al. 2023). Numerous studies have shown that levels of TNF‐α, IL‐1β, and IL‐6 are significantly elevated in the serum or bronchoalveolar lavage fluid of patients with acute pneumonia or in LPS‐induced animal models (Berkowitz et al. 2022; Del Valle et al. 2020). However, upon TNF‐α binding to its receptor TNFR, multiple proteases are activated, subsequently regulating the NF‐κB/MAPKs signaling pathways. Therefore, by inhibiting TNFR activity and blocking the NF‐κB and MAPKs signaling pathways, the severity of inflammation can be alleviated. TNF‐α has been identified as a key mediator of inflammation, inducing the secretion of inflammatory factors such as IL‐6 and IL‐1β, resulting in inflammatory responses following LPS pulmonary instillation (Van Loo and Bertrand 2023). In this study, LPS instillation in SD rats increased TNF‐α release, causing severe destruction of alveolar structures with inflammatory infiltration and significant collagen deposition, suggesting that LPS induced lung lesions through the production of TNF‐α. Treatment with various doses of ganodermanontriol reversed the pulmonary pathological changes in model rats and significantly inhibited the release of inflammatory factors. Molecular mechanism studies revealed that ganodermanontriol could inhibit the expression of TNFR and downstream NF‐κB/MAPKs signaling pathways, with the therapeutic effect in the high‐dose group being superior to that of the positive control drug dexamethasone. This study explored the anti‐inflammatory potential of ganodermanontriol in an acute pneumonia model. Existing research indicates that triterpenoids from the Ganoderma genus exhibit notable anti‐inflammatory and protective effects in various disease models. For instance, a formulation containing ganodermanontriol (0.0632%) successfully mitigated ethanol‐induced liver injury by modulating the MKP1/MAPK signaling pathway, demonstrating its significant role in inhibiting inflammation and tissue damage (Chen et al. 2023). Moreover, ganodermanontriol reduced LPS/D‐Galactosamine‐induced acute liver injury by inhibiting the TLR4‐MyD88‐mediated NF‐κB and MAPK signaling pathways, further verifying its multifunctional roles in anti‐inflammatory responses (Hu et al. 2020). Another significant finding related to this research is that the structurally similar ganodermanondiol can effectively inhibit melanin production through the MAPK cascade, indicating that such compounds may have extensive application potential in regulating signaling pathways (Kim et al. 2016). The similarity in action mechanisms implies that Ganoderma triterpenoids might exert wide‐ranging signal regulatory effects in various physiological processes. Additionally, the anti‐complement activity of ganodermanontriol has been well‐established, exhibiting significant immunomodulatory and anti‐inflammatory effects by inhibiting the classical pathway (CP) of the complement system (Min et al. 2001). Particularly, the inhibitory efficacy of ganodermanontriol is further increased by adding a hydroxymethyl group to its side chain structure. This observation suggests that the hydroxymethyl group is essential for its biological activity. Ganodermanontriol, as a naturally sourced triterpenoid compound, displays various biological activities through its unique chemical structure, particularly significant effects in anti‐inflammatory and immunomodulatory aspects. Structural modifications, such as introducing additional functional groups or altering existing ones, may further optimize its pharmacological properties, enhance efficacy and specificity, and reduce side effects, thereby increasing its potential for new drug development. This optimization could enable ganodermanontriol to play a broader role in treating inflammatory diseases, offering new directions and possibilities for future drug research and development.

## 5 Conclusions

Building on existing research, this study hypothesizes that ganodermanontriol can effectively alleviate pathological changes and inflammatory responses associated with acute pneumonia by modulating the phosphorylation and activation of the TNF/NF‐κB/MAPK signaling pathways. This hypothesis is consistent with the observed anti‐inflammatory and immunomodulatory effects of ganodermanontriol in other models. The study provides scientific evidence supporting the potential of ganodermanontriol in the treatment of acute pneumonia and offers new insights into the development of natural anti‐inflammatory agents for pneumonia management.

## Author Contributions

**Shizhan Deng:** software (lead), writing – original draft (lead). **Dequan Zhong:** software (lead), writing – original draft (lead). **Yonggan Dong:** data curation (supporting), formal analysis (lead). **Yanan Qian:** software (supporting), writing – original draft (supporting). **Biao Wang:** methodology (supporting). **Mengxue Hu:** validation (supporting). **Meng Liu:** validation (supporting). **Kemeng Tan:** formal analysis (supporting). **Chaojie Zhang:** data curation (equal). **Heng Tang:** funding acquisition (supporting), project administration (supporting), writing – review and editing (supporting).

## Ethics Statement

All the animal experiments were carried out at Wanbei Coal Electric Group General Hospital. All experiments were conducted in accordance with the principles of the Guide for the Care of Laboratory Animals and were approved by the Institutional Animal Ethics Committee (IAEC) of Wanbei Coal Electric Group General Hospital. This study followed the National Institutes of Health's Guide for the Care and Use of Laboratory Animals to the code. All of the operations were done under sodium pentobarbital anesthesia, and every attempt was made to reduce pain.

## Conflicts of Interest

The authors declare no conflicts of interest.

## Supporting information


Data S1.

## Data Availability

The data that support the findings of this study are available in the Supporting Information of this article. The data that support the findings of this study are available from the corresponding author upon reasonable request.
