# Supplementary material for: Network Pharmacology and Experimental Validation Reveal Ganodermanontriol Modulates Pneumonia via TNF/NF‐κB/MAPKs Signaling Pathway
Source: Food Sci Nutr. 2025 Mar 25;13(4):e70123. doi: 10.1002/fsn3.70123 (PMC11936839; doi:10.1002/fsn3.70123)
Supplement: Supplementary file 1 — Data S1. [file FSN3-13-e70123-s001.docx]

Supporting Information

**Network Pharmacology and Experimental Validation Reveal Ganodermanontriol Modulates Pneumonia via TNF/NF‑κB/MAPKs Signaling Pathway**

Shizhan Deng^a^, Dequan Zhong^a^, Yonggan Dong^a^, Yanan Qian^c^, Biao Wang^b^, Mengxue Hu^a^, Meng Liu^a^, Kemeng Tan^a^, Chaojie Zhang^a^, Heng Tang^a*^

a. Wanbei Coal Electric Group General Hospital, Anhui Province, Suzhou 234011.

b. Wanbei Coal Electric Group General Hospital Affiliated to Bengbu Medical University, Anhui Province, Suzhou 234011.

c. Department of Orthopedics and Traumatology, Orthopedic Trauma Faculty, Henan University of Chinese Medicine, Zhengzhou, Henan, China

* Corresponding author: Wanbei Coal Electric Group General Hospital, Anhui Province, Suzhou 234011. Tel/Fax: 3975031. Email: tangheng@mail.ustc.edu.cn

Shizhan Deng and Dequan Zhong contributed equally to this paper.

Table S1. Liquid phase conditions for HPLC of the ethanol extract of Ganoderma lucidum.

| Time（min） | Flow rate(ml/min) | Methanol（%） | 0.03%Acetic acid（%） |
| --- | --- | --- | --- |
| 0 | 1 | 50 | 50 |
| 3 | 1 | 50 | 50 |
| 5 | 1 | 68 | 32 |
| 30 | 1 | 82 | 18 |
| 40 | 1 | 87 | 13 |
| 55 | 1 | 91 | 9 |
| 65 | 1 | 95 | 5 |
| 70 | 1 | 100 | 0 |

Table S2. HPLC analysis of the constituents of *G. lucidum* triterpenes.

| No. | Retention time (min) | Compound name |
| --- | --- | --- |
| 1 | 18.75 | Ganoderiol J |
| 2 | 23.22 | Ganoderiol D |
| 3 | 31.39 | Ganoderiol A |
| 4 | 33.85 | Lucidadiol |
| 5 | 35.42 | Ganodermanontriol |
| 6 | 42.61 | Ganoderiol F |
| 7 | 50.84 | Ganoderiol B |
| 8 | 56.90 | Ganodermic acid DM |

Table S3 Hematological parameters of rat treated orally with the ganodermatriol in an acute toxicity test.

| Hematological parameters | Males Control | Males Gano | Females Control | Females Gano |
| --- | --- | --- | --- | --- |
| HGB (g/L) | 150.20 ± 8.40 | 149.30 ± 7.95 | 149.80 ± 5.90 | 151.30 ± 5.20 |
| WBC (×109/L) | 5.01 ± 1.60 | 4.50 ± 1.30 | 6.32 ± 2.05 | 5.40 ± 2.10 |
| RBC (×1012/L) | 8.45 ± 0.90 | 8.90 ± 0.42 | 8.10 ± 1.20 | 9.01 ± 0.50 |
| PLT (×109/L) | 950.4 ± 130.25 | 910.5 ± 135.10 | 740.6 ± 75.00 | 758.3 ± 79.20 |
| HCT (L/L) | 43.89 ± 4.75 | 46.23 ± 5.20 | 44.90 ± 6.50 | 48.12 ± 4.80 |
| MCH (pg) | 17.00 ± 0.35 | 16.95 ± 0.30 | 16.88 ± 0.33 | 16.92 ± 0.30 |

The values are expressed as means ± standard deviation (5 rats/sex/group). No significant differences between the control and treatment groups.

Table S4 Serum biochemistry of mice treated orally with the ganodermatriol in an acute toxicity test.

| Hematological parameters | Males Control | Males Gano | Females Control | Females Gano |
| --- | --- | --- | --- | --- |
| ALB (g/L) | 27.67 ± 2.08 | 25.13 ± 1.46 | 31.98 ± 1.44 | 31.58 ± 1.53 |
| ALT (U/L) | 59.10 ± 13.67 | 60.50 ± 8.30 | 72.90 ± 14.49 | 71.20 ± 14.86 |
| AST (U/L) | 267.10 ± 36.88 | 272.70 ± 37.96 | 270.90 ± 60.97 | 268.80 ± 65.00 |
| UREA (mmol/L) | 8.54 ± 1.26 | 8.30 ± 1.38 | 6.87 ± 1.16 | 7.78 ± 1.47 |
| CREA (µmol/L) | 7.83 ± 0.94 | 7.81 ± 0.21 | 4.70 ± 1.96 | 4.73 ± 1.75 |
| ALP (U/L) | 123.00 ± 22.40 | 145.70 ± 40.06 | 163.80 ± 43.99 | 147.30 ± 30.88 |
| CHOL (mmol/L) | 3.14 ± 0.44 | 3.22 ± 0.48 | 2.27 ± 0.43 | 2.39 ± 0.33 |

The values are expressed as means ± standard deviation (5 rats/sex/group). No significant differences between the control and treatment groups.


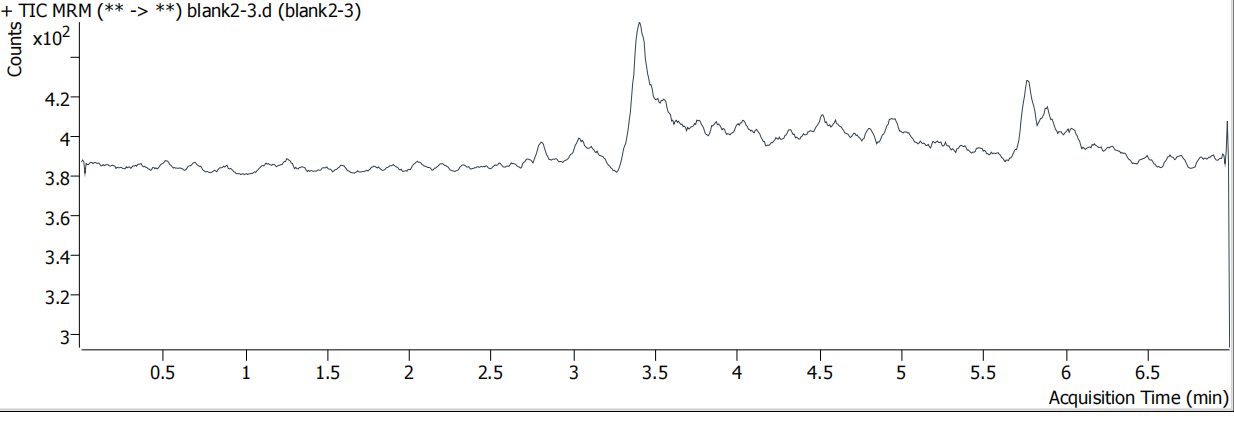


Figure.S1. Total ions chromatograms for quantitative analysis of four triterpenoids in GLA by LC-MS.


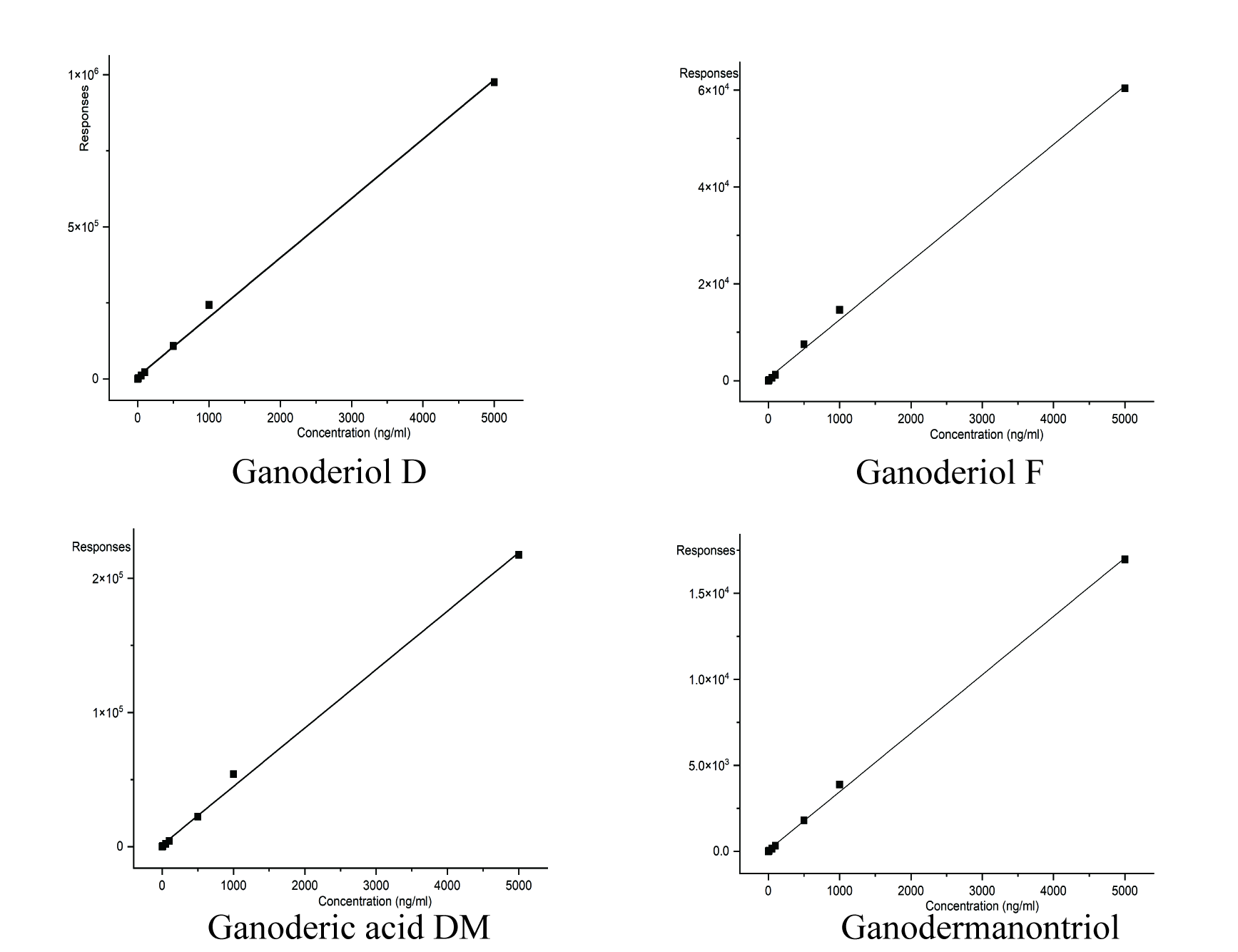


Figure S2. Standard curves for the quantification of four triterpenes by LC-MS.


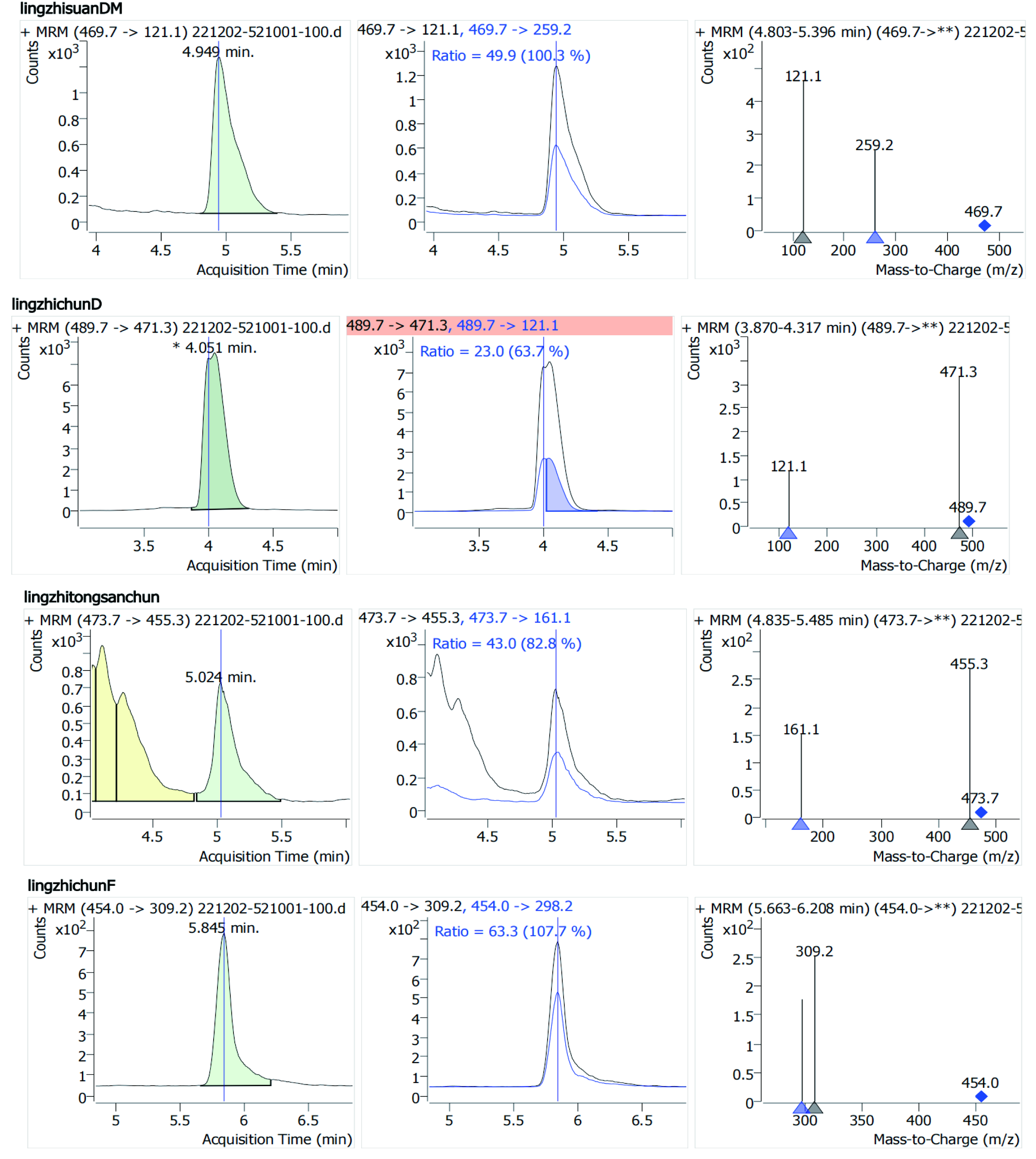


Figure S3. High-resolution mass spectra of four triterpenes.
